# Metabolic insights into HIV/TB co-infection: an untargeted urinary metabolomics approach

**DOI:** 10.1007/s11306-024-02148-5

**Published:** 2024-07-16

**Authors:** Cara Olivier, Laneke Luies

**Affiliations:** Focus Area Human Metabolomics, North-West University, Potchefstroom Campus, Private Bag X6001, Box 269, Potchefstroom, North West 2520 South Africa

**Keywords:** TB, HIV, Co-infection, Metabolomics, Urine, GC-MS

## Abstract

**Introduction:**

Amid the global health crisis, HIV/TB co-infection presents significant challenges, amplifying the burden on patients and healthcare systems alike. Metabolomics offers an innovative window into the metabolic disruptions caused by co-infection, potentially improving diagnosis and treatment monitoring.

**Aim:**

This study uses untargeted metabolomics to investigate the urinary metabolic signature of HIV/TB co-infection, enhancing understanding of the metabolic interplay between these infections.

**Methods:**

Urine samples from South African adults, categorised into four groups — healthy controls, TB-positive, HIV-positive, and HIV/TB co-infected — were analysed using GCxGC-TOFMS. Metabolites showing significant differences among groups were identified through Kruskal-Wallis and Wilcoxon rank sum tests.

**Results:**

Various metabolites (*n* = 23) were modulated across the spectrum of health and disease states represented in the cohorts. The metabolomic profiles reflect a pronounced disruption in biochemical pathways involved in energy production, amino acid metabolism, gut microbiome, and the immune response, suggesting a bidirectional exacerbation between HIV and TB. While both diseases independently perturb the host’s metabolism, their co-infection leads to a unique metabolic phenotype, indicative of an intricate interplay rather than a simple additive effect.

**Conclusion:**

Metabolic profiling revealed a unique metabolic landscape shaped by HIV/TB co-infection. The findings highlight the potential of urinary differential metabolites for co-infection, offering a non-invasive tool for enhancing diagnostic precision and tailoring therapeutic interventions. Future research should focus on expanding sample sizes and integrating longitudinal analyses to build upon these foundational insights, paving the way for metabolomic applications in combating these concurrent pandemics.

**Supplementary Information:**

The online version contains supplementary material available at 10.1007/s11306-024-02148-5.

## Introduction

In the global fight against infectious diseases, the human immunodeficiency virus (HIV) and tuberculosis (TB) are formidable adversaries, both declared as pandemics due to their widespread impact and endemic hurdles. Major hurdles encompass factors such as social stigmatisation, limited access to healthcare services, especially in poverty-stricken areas, and the significant economic burden imposed by these diseases. The emergence of drug resistance and the impact of concurrent pandemics, like COVID-19, further exacerbate the complexities of managing HIV and TB (Olivier & Luies, 2023). Despite extensive research and substantial investments aimed at control and eradication, these diseases continue to pose significant public health threats. In 2023 alone, there were approximately 1.5 million new HIV infections and significant AIDS-related mortality (Joint United Nations programme on HIV/AIDS, 2023). Meanwhile, TB, caused by *Mycobacterium tuberculosis* (*Mtb*), as a leading cause of death from a single infectious agent, was responsible for the deaths of a staggering 1.3 million people in the same year, excluding 167 000 deaths among HIV-positive individuals, highlighting the lethal interplay between these diseases. Notably, of the estimated 10.6 million people diagnosed with TB, only about 80% are aware of their HIV status, underscoring the crucial need for improved diagnostic capabilities and integrated health services (World Health Organization, 2023). This data underscores HIV and TB’s lethal impact globally and points to the critical need for improved diagnostic and management strategies.

The advent of the COVID-19 pandemic has further complicated the dynamics of disease management, particularly for TB, as health services faced unprecedented disruptions. This disruption led to a notable decline in TB case detection and treatment, highlighting vulnerabilities in our health systems and the urgent need for resilient healthcare solutions (World Health Organization, 2021). The co-infection of HIV and TB, especially in regions with high prevalence of both diseases, exacerbates these challenges, intensifying the strain on healthcare resources and necessitating innovative approaches to treatment and management (Olivier & Luies, 2023).

Globally, the syndemic of HIV and TB represents a dual threat that exacerbates the epidemiological and clinical complexities of each disease. The interplay between HIV and TB is particularly lethal; HIV compromises the immune system, significantly increasing the susceptibility to TB, which in turn can accelerate the progression of HIV-related symptoms and complicate the management of both infections (Debelu, 2022; Rewari et al., 2021). This dynamic underscores the need for multisectoral accountability in health responses and highlights the innovations in treating drug-resistant TB as critical components of future strategies. For instance, in regions like sub-Saharan Africa, this interrelationship is starkly evident with HIV-infected individuals facing a 16 to 27-fold increased risk of developing active TB disease (World Health Organization, 2023; Olivier & Luies, 2023).

Significant progress has been made, yielding valuable insights into better understanding the individual metabolic changes associated with HIV and TB through metabolomics. Metabolomics, the study of small molecule metabolites in biological samples, offers a promising avenue to explore these complex interactions (De Villiers & Loots, 2013; Herbert et al., 2023). Metabolomics has been instrumental in identifying biomarkers for diagnosis, treatment monitoring, and understanding the pathophysiology of these diseases (Liebenberg et al., 2021). For HIV, studies have highlighted alterations in lipid and energy metabolism, immune activation, oxidative stress pathways, as well as dysregulation of amino acid metabolism. Specifically, HIV infection is associated with dyslipidemia, characterised by elevated levels of triglycerides and low-density lipoprotein cholesterol, along with reduced high-density lipoprotein cholesterol levels. Energy metabolism disturbances include altered glucose metabolism and mitochondrial dysfunction, which contribute to increased oxidative stress and the production of reactive oxygen species (ROS) (Thirion et al., 2024; Rose et al., 2008). Additionally, HIV infection disrupts the gut microbiome, leading to dysbiosis, which further exacerbates systemic inflammation and immune activation (Vujkovic-Cvijin et al., 2013). These reflects the virus’ impact on host metabolism and immune function.

Similarly, TB metabolomics has revealed alterations in glycolysis, lipid profiles, amino acid utilisation, and markers of inflammation and oxidative stress. Specifically, TB results in elevated levels of tryptophan and its metabolites, such as kynurenine, which are involved in immune regulation and pathogen defence. Lipid metabolism is also profoundly affected, with increased levels of host-derived lipids, such as cholesterol and fatty acids, utilised by the pathogen for survival and persistence within host cells (Yu et al., 2023). Furthermore, TB is associated with increased inflammatory cytokines and oxidative stress markers, reflecting the host’s immune response to the pathogen. The gut microbiome in TB patients is often disrupted, with a decrease in beneficial commensal bacteria and an increase in pathogenic bacteria, contributing to disease progression and severity (Ghoshal et al., 2024). Understanding the pathogen’s adaptation strategies and host-pathogen interactions, could aid the discovery of potential diagnostic and prognostic biomarkers.

However, the metabolic repercussions of HIV/TB co-infection remain less explored and poorly understood as few studies have focused on the combined metabolic impact of these diseases. Understanding the integrated metabolic responses in co-infected individuals, how these responses are altered, and the distinct metabolic pathways affected, is crucial for advancing diagnostic accuracy, treatment optimisation, and patient management strategies. Metabolomics can provide such unique insights, potentially revealing novel biomarkers and therapeutic targets (Liebenberg et al., 2021). The importance of metabolomics is further highlighted by the integration of health services, including collaborative HIV/TB activities, which are essential for addressing the dual burden of these infections (World Health Organization, 2023). Recent serum-based research suggests that co-infection leads to distinct metabolic alterations that differ from those seen in either disease alone, pointing to unique pathophysiological interactions (Herbert et al., 2023). This underscores the need for further exploration into the metabolic consequences of HIV/TB co-infection to uncover novel biomarkers and therapeutic targets.

This investigation seeks to comprehensively characterise the urinary metabolic effects of HIV/TB co-infection using an untargeted GC-MS metabolomics approach. By aligning these metabolomic insights with a broader understanding of HIV and TB, we aim to lay the groundwork for comprehensive, multidisciplinary strategies to combat this dual threat. Ultimately, this exploratory study contributes to the metabolomics field by providing a foundational perspective for researchers exploring the complex challenges posed by HIV/TB co-infection, potentially guiding future research that could improve patient care and outcomes.

## Materials and methods

### Sample collection and demographics

Urine samples were collected under standardised protocols (Bi et al., 2020) by the South African Tuberculosis Vaccine Initiative (SATVI) and the Desmond Tutu HIV Foundation at the University of Cape Town. From here, the de-identified samples were transported to the North-West University’s Focus Area Human Metabolomics and stored at -80^o^C. Participants, aged 18–69 from Masiphumelele and Ocean View Townships in Cape Town (South Africa), were selected based on strict inclusion criteria to minimise *Mtb* strain variability and exclude potential confounding factors such as concurrent diseases, medication use, or pregnancy/lactation. This approach was important to ensure a more homogeneous study population, thereby enhancing the robustness of our findings related to HIV and TB co-infection.

Participants were categorised into four groups based on serum-confirmed HIV and TB status, diagnosed using GeneXpert for TB and dual rapid antibody tests for HIV, following South African/World Health Organization (WHO) guidelines. No post-hoc anonymous HIV testing was conducted on the samples. All participants were treatment naïve, and those testing positive for HIV were referred for appropriate healthcare services. The “HIV-/TB-” cohort consisted of healthy, asymptomatic subjects. Cohort demographics are available in Table 1, with CD4 T cell counts and viral loads only recorded for HIV-positive individuals. Notably, these samples were shared by local township clinics for research purposes (with ethical approval and consent obtained); the clinics do not routinely test CD4 and viral load when diagnosing HIV, in accordance with WHO guidelines. The WHO guidelines for HIV diagnosis stipulate two consecutively positive HIV tests rather than routine CD4 and viral load testing.

**Table 1:** Cohort demographics and clinical data

	HIV-/TB-	HIV-/TB+	HIV+/TB-	HIV+/TB+
No. of patients (%)	32 (35.2)	41 (45.1)	9 (9.9)	9 (9.9)
Age (years), mean ± SD (range)	33.7 ± 11.2	35.3 ± 12.2	37 ± 5.3	33.4 ± 5.8
Sex, male: female ratio	20:12	10:31	4:5	3:6
Smokers in the group (%)	12 (37.5)	25 (61)	3 (33.3)	3 (33.3)
CD4 + T cell count (cells/mm^3^ blood), mean ± SD	N/A	N/A	269^#^ ± 119.4	135.4 ± 140.2
Viral load (copies/mm^3^), range*	N/A	N/A	Unavailable	< 20–124

*Abbreviations: HIV: human immunodeficiency virus; TB: tuberculosis; -: negative; +: positive; SD: standard deviation; CD4: cluster of differentiation 4; N/A: not applicable. Groups: HIV-/TB-: healthy controls; HIV-/TB+-: untreated TB-positive patients; HIV+/TB-: untreated HIV-positive patients; HIV+/TB+: untreated HIV/TB co-infected patients*

*Since the viral load was not available for all patients,* the data shown here do not represent the entire group*

*#This average is based on only two samples for which this data was available*

Given the small sample sizes, particularly for HIV-positive (*n* = 9) and HIV/TB co-infected (*n* = 9) groups, due to the WHO’s “test-and-treat” policy (World Health Organization, 2021), the study’s explorative nature aimed to guide future research rather than directly discovering new biomarkers.

### Reagents and chemicals

3-Phenylbutyric acid, methoxamine hydrochloride (MOX-HCl), and N,O-bis(trimethylsilyl)trifluoroacetamide (BSTFA) with 1% trimethylchlorosilane (TMCS) were from Sigma Aldrich (St. Louis, Missouri, USA). Acetonitrile was from Burdick and Jackson (Honeywell International Inc., Muskegon, USA), while pyridine was purchased from Merck (Darmstadt, Germany).

### Sample extraction and derivatization

Quality control (QC) samples, system blanks (SBs), and extraction blanks (EBs) are critical components of metabolomics analyses aimed at ensuring data quality and integrity. QC samples were prepared by pooling a small volume from all collected urine samples into a single vial, which was used as a reference standard throughout the analysis. These QC samples serve to (i) condition the analytical platform, (ii) measure intra-study reproducibility, and (iii) correct for systematic errors. SBs and EBs are employed to assess and minimise sources of contamination and background noise in untargeted metabolomics. SBs involve running a “blank” gradient with no sample or reagents to identify any impurities in the separation system. EBs, on the other hand, mimic the sample preparation process without any biological sample, thereby revealing contaminants introduced during sample handling and processing. These blank samples are crucial for identifying and removing signals from non-biological sources that could otherwise confound the data interpretation (Broadhurst et al., 2018).

Additionally, dividing many samples into batches over consecutive days is necessary to manage the analytical workload. By analysing samples in batches along with quality assurance samples, researchers can detect and mitigate potential batch effects, maintaining data quality and reducing the impact of analytical variability on the results. In this study, each analytical batch represented the entire cohort, and included a systematic arrangement of SBs, EBs, QCs, and experimental samples.

Samples were analysed as described by Olivier et al. (2023). Briefly, to each 100 µL urine sample, 100 µL of 3-phenylbutyric acid (50 ppm) as the internal standard was added, followed by the addition of ice-cold acetonitrile (300 µL). The mixture was then centrifuged at room temperature (15 000 *x* g for 5 min), and the resulting supernatant was carefully transferred into a GC vial. The samples were dried under a stream of nitrogen gas at 37 °C. For methoximation, 50 µL of MOX-HCl in pyridine (20 mg/mL) was added, followed by vortexing for 30 s, and incubation for 90 min at 50 °C. After a second drying step under the same conditions, silylation was performed using 50 µL of BSTFA with 1% TMCS for 60 min at 60 °C. The prepared samples were then placed into glass inserts, transferred to GC vials, and securely capped for analysis.

### GC-MS analysis

GCxGC-TOFMS analysis was conducted as described in Olivier et al. (2023). In summary, following a routine instrument maintenance check, samples, along with QCs and blanks, were analysed in seven randomised batches using a Pegasus 4D GCxGC-TOFMS system with an Agilent 7890 GC, employing a dual-column setup for chromatographic separation, with a Restek Rxi-5MS primary capillary column (28.2 m; 250 μm diameter; 0.25 μm film thickness), and a Restek Rxi-17 secondary capillary column (1.3 m; 250 μm diameter; 0.25 μm film thickness). Each batch sequence was as follows: SB1; fatty acyl methyl esters (FAMEs; used as retention index standards); QC1 (injected thrice to prime the analytical platform); EB1; randomised patient samples; QC2; EB2; randomised patient samples; QC3; SB2; EB3; randomised patient samples; QC4; EB4; randomised patient samples; EB5; QC5; SB3; and FAMEs.

Samples (1 µL) were injected using a 1:10 split ratio with purified helium as a carrier gas, set at a constant flow of 1.4 mL/min. The front inlet temperature was maintained at 250 °C, the transfer line at 225 °C, and the ion source at 200 °C. Cryomodulation was achieved with a hot pulse of nitrogen gas for 0.7 s, every 3 s. The primary oven programme started at 70 °C, which was held for 1 min. Hereafter, the temperature was ramped as follows: 5 °C/min to 100 °C, 10 °C/min to 160 °C, 13 °C/min to 230 °C, and finally 20 °C/min to 300 °C, where it was maintained for 2 min. The secondary oven was programmed identical to that of the primary oven, except for a + 5 °C at each ramping interval. The total run time lasted approximately 33 min per sample, with a solvent delay of 480 s before data acquisition. Mass spectra were collected from 50 to 800 *m/z* at an acquisition rate of 20 spectra per second, with optimised detector settings (Olivier et al., 2023).

The data was processed using LECO Corporation’s ChromaTOF software (version 4.32). This involved deconvolution to differentiate co-eluting peaks by their distinct spectra, peak alignment to ensure consistency across different sample runs, and peak identification (Du & Zeisel, 2013; Issaq et al., 2009). The peak alignment parameters included an expected peak width of 3 s for the first dimension and 0.1 s for the second dimension and adjusting the signal-to-noise ratio to 300 with a minimum of 2 apexing peaks. For peak identification/annotation, spectra were matched against in-house libraries, compiled from previously injected standards, and the NIST (version 2.2) mass spectral libraries (mainlib, replib), requiring at least a 65% similarity match prior to visual inspection (see Sect. 3.1). Notably, the FAMEs injections were visually inspected to check for retention time (RT) drift and confirmed the retention indexing of compounds. Duplicate peaks were combined following visual inspection if the compound annotation, unique mass, and RT matched.

### Data cleanup

Initially, SB and EB corrections were made as previously described to identify and remove signals from non-biological sources that could potentially confound the interpretation of results (Broadhurst et al., 2018). Next, the data was normalised making use of mass spectral total useful signal (MSTUS) (Warrack et al., 2009) and applying creatinine normalisation by dividing with each sample’s pre-determined creatinine value. Hereafter, a 50% quality control-coefficient of variation (QC-CV) filter was applied by eliminating compounds with CV values greater than 50%, indicating excessively fluctuating compounds (Luies & Loots, 2016). The data was also checked for batch effects (Supplementary Fig S1); however, no significant batch effects were present when considering the QC samples, demonstrating a consistent instrumental response and stable column performance throughout the analysis. Following this, a 50% zero filter was applied to remove spurious or infrequently detected compounds that could introduce variability or noise into the results (Smuts et al., 2013). This threshold helps ensure that only metabolites consistently present across most samples are included in the analysis, thereby enhancing the reliability of the findings. Following these cleanup steps, the initial dataset of 663 compounds, including both annotated and unannotated features, was refined to 334 compounds for univariate and multivariate statistical analysis (see Supplementary Fig S2). Non-derivatized compounds were not removed because our untargeted approach aimed to include all detected features for comprehensive analysis and coverage of the metabolomic landscape associated with HIV/TB co-infection. Identification of metabolites relied on spectral matching and library annotations, which may not be completely certain without standards for verification. Thus, such inclusion allows for further investigation of potentially novel or misannotated metabolites.

### Statistical analysis

Prior to statistical tests, the data underwent log-transformation and autoscaling to assume a (relatively) normal distribution, a crucial prerequisite condition for many statistical tests (Field, 2013). Both univariate and multivariate analyses (Saccenti et al., 2014) were applied using MetaboAnalyst (version 5.0).

Univariate analysis included the Wilcoxon rank sum and Kruskal-Wallis tests. The Wilcoxon rank sum test was employed to compare the distribution of metabolic profiles between each of the diseased groups (i.e., HIV-positive, TB-positive, and HIV/TB co-infection) and healthy controls independently (Hollander et al., 2013). This approach was chosen as it is effective in handling non-parametric data, especially when comparing median values in datasets that do not necessarily follow a normal distribution. Also, a key component of our statistical analysis was the non-parametric Kruskal-Wallis test, an alternative to the one-way analysis of variance (ANOVA), used for comparing multiple independent groups. This test is apt for our study as it is ideal for small sample size and data that is not normally distributed or is ordinal (Taylor, 2023). Our data fits these criteria given its small sample size and skewed distribution.

Multivariate analyses included principal component analysis (PCA) and partial least squares discriminant analysis (PLS-DA). These methods allow for an integrated view of the data, highlighting patterns and relationships that are not apparent in univariate analysis. PCA was utilised to condense the multivariate data, simplifying the complex data structure into a more manageable form. This method is invaluable for identifying patterns, trends, or outliers in the data (Eriksson et al., 2013). PLS-DA was employed as a method of discriminant analysis, useful for classifying observations into predefined groups based on their metabolic profile (Saccenti et al., 2014; Eriksson et al., 2013).

## Results

### Univariate testing

The Kruskal-Wallis test was employed to compare differences across the four groups: TB-positive (HIV-/TB+), HIV-positive (HIV+/TB-), co-infected (HIV+/TB+), and healthy controls (HIV-/TB-). This test identified 15 urinary metabolic compounds (see Supplementary Fig S3, Fig S4, and Table S1) affected by a complex interplay of various factors, reflecting combined metabolic pathways rather than a single cause.

Although the Kruskal-Wallis test identified significant metabolites indicating differences among the four groups, it did not specify which particular groups differed. To address this limitation, subsequent pairwise comparisons using the Wilcoxon rank sum test was conducted, pinpointing specific metabolites that varied between groups. This non-parametric test is particularly suitable when data is not normally distributed (McKight & Najab, 2010). Detailed results of these pairwise comparisons are available in Supplementary Table S2.

This exploratory proof-of-concept study takes a holistic approach in analysing metabolite variations, linking results from both the Kruskal-Wallis and Wilcoxon rank sum tests to provide a comprehensive snapshot of the significant metabolic differences observed among the study groups (Table 2). This method differs from more targeted studies as it encompasses a broader range of metabolites, designed to guide future research. The metabolomics standards initiative (MSI) confidence level of identification (Fiehn et al., 2007), as well as the library similarity match have also been indicated in Table 2 (see Supplementary Fig S5 for spectral matching).

**Table 2:** Significant metabolite summary, based on univariate approaches

Metabolite	*Kruskal-Wallis test results (Table S1)*: Group with highest average concentration	*Wilcoxon rank sum test result (Table S2):* Trend and comparison	MSI confidence level and similarity match
**Amino Acids and Related Metabolites**			
3-Phenyllactic acid	-	HIV-/TB+ ↑ versus HIV-/TB-	Level 2; 83.7%
Hydracrylic acid	HIV+/TB-	HIV-/TB+ ↓ versus HIV-/TB-	Level 2; 92.4%
Quinolinic acid	HIV+/TB+	HIV-/TB+ ↑ versus HIV-/TB-	Level 2; 93.0%
Tiglylglycine	HIV+/TB-	-	Level 2; 88.0%
Tyrosine	-	HIV-/TB+ ↑ versus HIV-/TB-	Level 2; 82.8%
**Carbohydrates**,** TCA cycle**,** and Energy Metabolism**			
Citric acid	HIV+/TB-	HIV-/TB+ ↓ versus HIV-/TB-	Level 2; 88.8%
Erythritol	HIV+/TB-	HIV-/TB+ ↓ versus HIV-/TB-	Level 2; 93.9%
Gluconolactone	HIV-/TB-	HIV-/TB+ ↓ versus HIV-/TB-	Level 2; 86.9%
Ribitol	-	HIV-/TB+ ↓ versus HIV-/TB-	Level 2; 82.5%
**Dicarboxylic Acids and Lipids**			
2-Methylene-butane-1,4-diol	HIV+/TB-	HIV-/TB+ ↑ versus HIV-/TB-	Level 2; 69.0%
3-Hydroxy-2-methylpropanoic acid	HIV+/TB-	HIV-/TB+ ↓ versus HIV-/TB-	Level 2; 86.6%
Methylmalonic acid	HIV+/TB-	-	Level 2; 91.2%
Methylsuccinic acid	-	HIV-/TB+ ↓ versus HIV-/TB-	Level 2; 88.5%
Octanoic acid	-	HIV-/TB+ ↓ versus HIV-/TB-	Level 2; 84.1%
Pentanedioic acid	HIV+/TB-	HIV+/TB- ↑ versus HIV-/TB-	Level 2; 84.4%
**Gut Microbiota Imbalance**			
2,3-Butanediol	HIV+/TB-	-	Level 2; 80.2%
2,3-Dihydroxybutanoic acid	HIV+/TB-	HIV-/TB+ ↓ versus HIV-/TB-	Level 2; 85.7%
5-Hydroxyvaleric acid	-	HIV-/TB+ ↓ versus HIV-/TB-	Level 2; 90.0%
Hippuric acid	HIV-/TB-	HIV-/TB+ ↓ versus HIV-/TB-	Level 2; 95.5%
**DNA Damage and Oxidative Stress**			
2-Deoxyribolactone	HIV+/TB-	-	Level 2; 84.6%
**Dietary Origin**			
2-Hydroxycyclohexane-1-carboxylic acid	-	HIV-/TB+ ↑ versus HIV-/TB-	Level 3; 67.6%
Diaveridine	HIV+/TB+	HIV-/TB+ ↑ versus HIV-/TB-	Level 3; 67.4%
**Exogenous Compounds**			
Ephedrine	-	HIV-/TB+ ↑ versus HIV-/TB-	Level 2; 83.8%

Abbreviations: HIV: human immunodeficiency virus; TB: tuberculosis; -: negative; +: positive; MSI: metabolomics standards initiative; ↑: increased; ↓: decreased

### Multivariate testing

We explored the use of PCA and PLS-DA to assess the relationships between different groups, specifically HIV-positive, TB-positive, and HIV/TB co-infection. The outcomes of these tests are presented in Fig S6.

PCA was employed to explore the inherent structure of the data, which did not show clear separation between the groups, indicating limited utility for further metabolite marker selection. Although PLS-DA provided clearer group separation, its use is subject to the outcomes of rigorous cross-validation and permutation testing. Cross-validation (Fig S7) revealed limitations in the model’s predictive accuracy, and poor permutation test results (Fig S8) further suggested inadequate predictive capacity. These findings imply that the observed group differences might not be statistically significant, potentially due to the small cohort size, as a larger sample size is generally required for a reliable estimation of empirical p-values. Consequently, these multivariate approaches were not relied upon for identifying significant differential metabolites due to concerns about overfitting and general reliability.

As noted by Saccenti et al. (2014), rigorous model validation is crucial to avoid overfitting and ensure generalisability in multivariate analysis. Due to the challenges and small sample size, we opted for univariate methods, which provided more robust and interpretable outcomes. The correct statistical tests and validation procedures were employed to mitigate the risks of false discoveries. Saccenti et al. (2014) highlight several reasons for discrepancies between multivariate and univariate results: (i) uninformative variables can mask significant information, (ii) accurate estimation of covariances and correlations is difficult with small sample sizes, and (iii) univariate and multivariate testing procedures may not always overlap. Therefore, while multivariate methods offer valuable insights, their limitations in our specific study justified the reliance on univariate approaches for more reliable results.

## Discussion

**Fig. 1:**
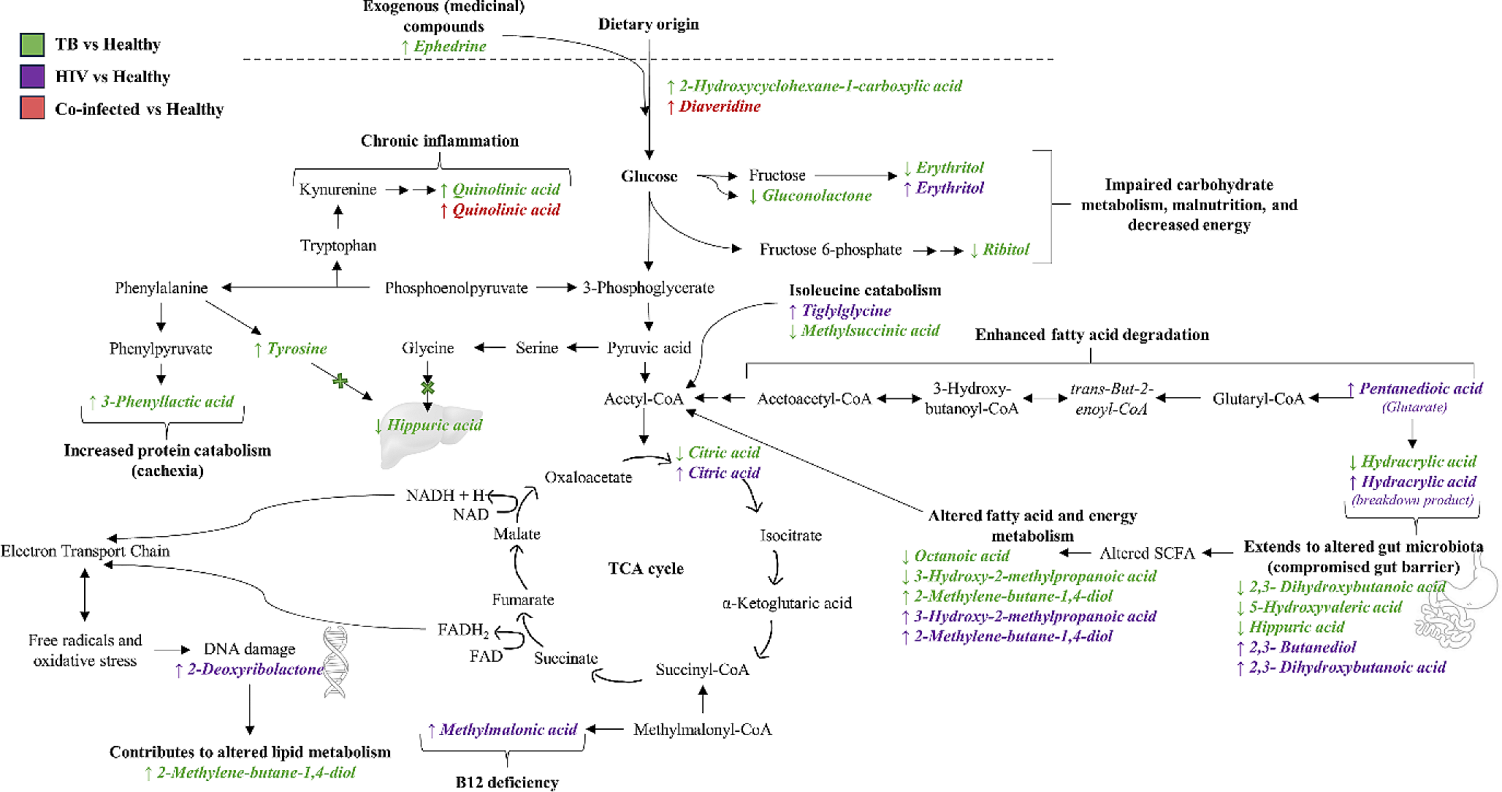
Metabolic alterations induced by HIV, TB, and co-infection. The figure illustrates the significant increases (↑) and decreases (↓) in metabolite levels observed when comparing healthy controls to patients with HIV (purple), TB (green), and HIV/TB co-infection (red)

In line with the study’s exploratory nature, the focus was directed toward the differential metabolites identified using the Kruskal-Wallis (*n* = 15; Table S1) and Wilcoxon rank sum (*n* = 23; Table S2) tests. Across the cohorts studied, 23 differential metabolites were discerned (Table 2; Fig. 1), each offering insights into the metabolic disruptions associated with HIV, TB, and co-infection. The ensuing discussion synthesises these metabolic anomalies, grounding them in biological relevance and offering fresh insights into the complex pathophysiological impacts of these diseases.

### Amino acids and related metabolites

Amino acids and related metabolites serve as critical indicators of the metabolic strain exerted by HIV and TB. Elevated 3-phenyllactic acid and tyrosine in HIV-/TB+ patients when compared to healthy controls, signal increased protein catabolism, potentially linked to the muscle wasting seen in cachexia (Luies & Du Preez, 2020; Luies & Loots, 2016; Amalia et al., 2022). The HIV+/TB- group’s raised tiglylglycine levels may be indicative of the body’s adaptive defence against chronic inflammation and oxidative stress (Aguayo-Cerón et al., 2023). Additionally, the virus’ impact on liver function, pivotal in amino acid metabolism, could be a contributing factor, as well as the nutritional status of these individuals, affecting the absorption and metabolism of various nutrients (Zhang et al., 2023). Notably, the increase in pentanedioic acid (resulting from amino acid degradation) and hydracrylic acid [a breakdown product of branched-chain amino acids and propanoic acid (Wishart et al., 2022)] among HIV+/TB- patients suggests disruptions extending into fatty acid metabolism and an altered gut microbiota (Gholson et al., 1959; González-Hernández et al., 2019). Furthermore, quinolinic acid was increased in the HIV-/TB+ and HIV+/TB+ groups, highlighting the compounding effect of the diseases on chronic inflammatory response pathways, with co-infection leading to even more severe inflammation and potential neurological damage or dysfunction (Luies & Loots, 2016; Blumenthal et al., 2012). These findings affirm that HIV and TB each uniquely distort host metabolism, with co-infection amplifying these effects, warranting further exploration into these metabolites as potential diagnostic differential metabolites.

### Carbohydrates, TCA cycle, and energy metabolism

In the realm of carbohydrates and the tricarboxylic acid (TCA) cycle, the metabolic shifts become even more pronounced. When compared across all groups, HIV’s influence is marked by an increase in citric acid, a key TCA cycle intermediate, reflecting the virus’ role in ramping up energy production to meet heightened metabolic demands and manage oxidative stress (Patil et al., 2019; Koppensteiner et al., 2012; Infantino et al., 2011). On the other hand, TB’s impact is starkly different, characterised by decreased levels of citric acid, ribitol, and gluconolactone, highlighting impairments in carbohydrate metabolism and energy synthesis. The observed decreases in ribitol and gluconolactone in HIV-/TB+ patients are particularly telling. Ribitol, a key component of riboflavin (vitamin B2) and flavin mononucleotide, plays a crucial role in the pentose phosphate pathway, which is integral to nucleotide and energy metabolism (Badolia et al., 2020). In TB, the disruption of this pathway may reflect a broader compromise in glucose metabolism, a common issue seen in HIV-/TB + patients who often exhibit diabetes-like symptoms (Luies & Loots, 2016). The highest concentrations of gluconolactone, an oxidised derivative of glucose, were observed in healthy controls, suggesting an unaltered glucose metabolism. This is interesting since the HIV-/TB+ patients showed a significant decrease in gluconolactone, further underscoring the metabolic stress exerted by TB. This stress could stem from TB’s known association with insulin resistance or a compromised insulin secretion (Luies & Loots, 2016), which not only affect glucose utilisation and energy production but also impact the body’s overall metabolic balance. This association between TB and altered glucose metabolism highlights the potential for TB to exacerbate or possibly precipitate diabetes-like conditions in affected individuals, adding another layer to the complex metabolic challenges posed by this infection. Additionally, erythritol levels decreased in HIV-/TB+ patients, suggesting disrupted sugar alcohol metabolism, which likely reflects the systemic effects of the disease on glucose homeostasis and overall metabolic health (Bisht et al., 2023). In contrast, the HIV+/TB- group exhibited the highest erythritol levels, possibly relating to HIV’s impact on glucose metabolism, notably through increased insulin resistance and altered hepatic glucose production, which can disrupt normal metabolic processes. Moreover, the oxidative stress induced by HIV may elevate erythritol levels as part of the body’s antioxidant response, serving as a compensatory mechanism to counteract the infection’s oxidative burden (Ivanov et al., 2016). This divergent response underscores the unique metabolic challenges presented by each infection and highlights the complex interplay of metabolic pathways influenced by HIV and TB.

### Dicarboxylic acids and lipids

The study further highlighted significant shifts in dicarboxylic acids and lipids, which are essential for energy production, signalling, and structural integrity within cells. In HIV-/TB+ patients, there was a marked decrease in octanoic acid and 3-hydroxy-2-methylpropanoic acid, indicating disruptions in lipid metabolism often associated with nutritional deficiencies and systemic metabolic stress caused by the disease. Notably, these metabolites are crucial for energy metabolism and hormone regulation, and its reduction could relate to TB’s impact on metabolic balance and hormonal function (Chang et al., 2013; Yurt et al., 2013). 3-Hydroxy-2-methylpropanoic acid also has a potential role in protein synthesis, possibly linking this metabolite to cachexia. A disrupted lipid metabolism is also indicated by the increased 2-methylene-butane-1,4-diol detected in the this group (Funderburg & Mehta, 2016).

Conversely, 3-hydroxy-2-methylpropanoic acid was found to be elevated in HIV+/TB- patients, suggesting that HIV-related changes, possibly gut dysbiosis, might increase the production of this short-chain fatty acid (SCFA) (Ranjbar et al., 2021; González-Hernández et al., 2019). This alteration could be a response to the immune system’s attempt to modulate inflammation and energy usage in the face of viral infection, as it can be converted to acetoacetyl-CoA and enter the TCA cycle. Additionally, the HIV+/TB- group showed increased levels of 2-methylene-butane-1,4-diol, likely linked to perturbations in lipid metabolism due to heightened inflammation (Funderburg & Mehta, 2016), as well as increased methylmalonic acid, indicative of nutrient absorption issues and B12 deficiency often observed in HIV-infected individuals (Boutin et al., 2022). These findings illustrate the distinct metabolic challenges imposed by each infection on dicarboxylic acids and lipids, reflecting the specific pathophysiological mechanisms of HIV and TB, and their combined impact in co-infected individuals.

### Gut microbiota imbalance

The interplay between TB, HIV, and the gut microbiota emerged as a significant factor in this study, highlighting profound systemic effects that extend beyond the primary infection sites. TB significantly alters the gut microbiome, leading to decreased diversity and abundance of beneficial bacterial populations (Luies & Du Preez, 2020; Winglee et al., 2014; Maji et al., 2018), impacting the production of 2,3-dihydroxybutanoic acid and key SCFAs such as 3-hydroxy-2-methylpropanoic acid and 5-hydroxyvaleric acid. SCFAs are crucial for maintaining gut integrity and modulating the immune system (Liu et al., 2021; Li et al., 2008; Wishart et al., 2022). Reduced SCFAs contribute to the aforementioned cachexia, often observed in TB patients (Das et al., 2015; Luies & Loots, 2016), correlating with poor treatment outcomes (Luies et al., 2017). Notably, these SCFA reductions were most pronounced in HIV-/TB+ patients compared to healthy controls, indicating greater gut microbiota function in healthy individuals. In HIV-/TB+ patients, reduced SCFA levels suggest a compromised gut barrier and an impaired immune response, potentially exacerbating the disease’s systemic impact. Furthermore, disturbances in the gut microbiota are reflected in decreased hippuric acid levels observed in the HIV-/TB+ patients (Mason et al., 2016). Hippuric acid (benzoyl glycine), a key urinary metabolite, traditionally forms from the conjugation of benzoate with glycine in the liver. Phenolic amino acids such as phenylalanine and tyrosine can contribute to the synthesis of hippuric acid through an alternative pathway involving their catabolism to phenylpyruvate and hydroxyphenylpyruvate, respectively, which can then be converted to benzoate (Pero, 2010). Interestingly, tyrosine was found to be increased in the HIV-/TB+ cohort (see Sect. 4.1), suggesting that impaired conjugation steps, possibly induced by chronic inflammation and altered enzyme activity or substrate availability, may contribute to the decreased hippuric acid excretion despite elevated precursor levels. To this end, the decreased hippuric acid excretion may also indicate liver dysfunction (Kohlstaedt & Helmer, 1936), linked to TB’s complex hepatic manifestations such as diffuse involvement, granulomatous hepatitis, or localised tuberculoma (Sonika & Kar, 2012), which can also adversely affect treatment outcomes (De Villiers & Loots, 2013). These insights underscore the intricate interplay between microbial metabolism, amino acid pathways, and hepatic function in shaping the urinary metabolic profile observed in TB patients. Future studies should explore the specific contributions of gut microbiota dysbiosis and metabolic alterations to the synthesis and excretion of hippuric acid as a potential diagnostic marker for TB and related metabolic disturbances (Mason et al., 2016).

In contrast, HIV’s impact on the gut microbiota leads to increased levels of 2,3-butanediol and 2,3-dihydroxybutanoic acid, reflecting disruptions to the gut epithelial barrier and a shift toward microbial populations favouring fermentative processes (Mills & Walker, 1989; Dillon et al., 2016). These changes are indicative of HIV’s broader influence on systemic inflammation and gut microbiome stability, which can exacerbate the host’s vulnerability to opportunistic infections and complicate treatment outcomes.

These findings highlight the critical role of the gut microbiota in the pathogenesis of both HIV and TB and underscore the potential for therapeutic strategies targeting microbiome restoration and stabilisation as part of comprehensive disease management.

### DNA damage and oxidative stress

Oxidative stress and DNA damage are critical factors in the progression of HIV and TB, with both conditions amplifying ROS production. In HIV, this oxidative stress leads to significant DNA damage, facilitating viral integration into the host genome and impacting the efficacy of antiretroviral therapy (Ivanov et al., 2016). 2-Deoxyribolactone, indicative of oxidative DNA damage (Chan et al., 2010), is notably elevated in HIV+/TB- individuals, reflecting the severe oxidative challenges they face. Similarly, TB-driven chronic inflammation also heightens oxidative stress (Luies & Du Preez, 2020), contributing to cellular and molecular damage. Effective management of oxidative stress could thus enhance treatment outcomes for both diseases, and in cases of co-infection.

### External influences

The study highlights significant external influences on the metabolic profiles of these patients, particularly from dietary sources and environmental exposures. The elevated levels of 2-hydroxycyclohexane-carboxylic acid, often found in foods and natural products (Wishart et al., 2022), reflect the unique dietary habits or the consumption of traditional medicines within the HIV-/TB+ cohort. Similarly, increased levels of diaveridine, an antibacterial agent used in animal feeds (Wang et al., 2022), suggest dietary exposure through animal products, with the highest levels in the HIV+/TB+ group indicating altered excretion and processing due to the compounded effects of both HIV and TB.

Ephedrine, used as a decongestant and bronchodilator (Limberger et al., 2013), was significantly elevated in HIV-/TB+ patients, highlighting its use to manage respiratory symptoms associated with TB. The presence of ephedrine indicates potential oversight in medication disclosure by participants, which could inadvertently influence the study’s metabolic findings.

These external factors substantially contribute to the observed metabolic variations, emphasising the importance of considering these influences when analysing metabolomic data. Understanding these external impacts is crucial for accurately interpreting metabolic changes and could inform more targeted therapeutic interventions and dietary recommendations for managing HIV, TB, and co-infection.

The findings from this study provide valuable insights into the metabolic alterations associated with HIV/TB co-infection, confirming the complex interplay between these two diseases. However, it is essential to acknowledge the limitations of our study, primarily regarding the generalisability of our findings. Despite efforts to match demographic factors and exclude significant comorbid conditions, the small sample size and the exploratory nature of this study inherently limit our ability to control for all potential confounding factors. This study is designed as a proof-of-concept to identify potential metabolic changes associated with HIV/TB co-infection. Moving forward, future studies should aim to collect additional clinical data, such as BMI, to provide a more comprehensive understanding of the metabolic alterations associated with HIV/TB co-infection. Additionally, studies with larger sample sizes and more rigorous matching criteria are necessary to validate these findings and further elucidate the metabolic interactions between HIV and TB.

## Conclusion

This study has unveiled distinctive metabolic profiles associated with HIV, TB, and their co-infection, providing new insights into the complex metabolic alterations induced by these diseases. By employing an untargeted GC-MS metabolomics approach, we identified specific urinary metabolites that reflect the significant biochemical shifts occurring in response to these infections. Our findings reveal that HIV and TB each uniquely distort host metabolism — HIV primarily influencing amino acid and nucleotide metabolism, and TB predominantly affecting lipid metabolism and the TCA cycle. Notably, the co-infected state does not merely combine the metabolic signatures of HIV and TB; rather, it reveals a unique metabolic milieu, suggesting a complex interplay that warrants further exploration.

The metabolites identified herein could serve as a foundation for developing non-invasive biomarkers for early detection and monitoring of disease progression and treatment response. However, our study also acknowledges the inherent limitations of small sample sizes and the exploratory nature of the research, which precludes definitive conclusions. These findings should serve as a springboard for larger-scale studies aimed at validating the identified metabolites and elucidating their roles in disease mechanisms.

Future research should aim to expand cohort sizes, integrate longitudinal sampling, and explore the impact of treatment regimens on metabolic profiles. Additionally, the integration of these metabolomic data with genomics and proteomics could offer a systems biology approach to comprehensively understand the multi-layered effects of HIV/TB co-infection. Through such integrative efforts, the goal of personalised medicine in the management of these pandemics may become a tangible reality, leading to more effective and targeted therapeutic interventions.

Ultimately, this research contributes to a growing body of knowledge that underscores the importance of metabolomics in infectious disease research. By delving into the metabolic consequences of HIV/TB co-infection, we can begin to piece together the intricate puzzle of these diseases, paving the way for advances in diagnostics, therapeutics, and our overall understanding of their global health impact.

### Electronic supplementary material

Below is the link to the electronic supplementary material.

Supplementary Material 1

## Data Availability

All data that form part of this paper and its supplementary material are free to obtain. Datasets are available on Biostudies, using accession number S-BSST1397.
