# Supplementary material for: Metabolic insights into HIV/TB co-infection: an untargeted urinary metabolomics approach
Source: Metabolomics. 2024 Jul 16;20(4):78. doi: 10.1007/s11306-024-02148-5 (PMC11252185; doi:10.1007/s11306-024-02148-5)
Supplement: Supplementary file 1 — Supplementary Material 1 [file 11306_2024_2148_MOESM1_ESM.pdf]

<u>**Supplementary Material:**</u>

Metabolic Insights into HIV/TB Co-infection: An Untargeted Urinary Metabolomics Approach

Cara Olivier and Laneke Luies*

Human Metabolomics, North-West University (Potchefstroom Campus), Potchefstroom, North West, South Africa

*Address correspondence to Laneke Luies: laneke.luies@nwu.ac.za

---

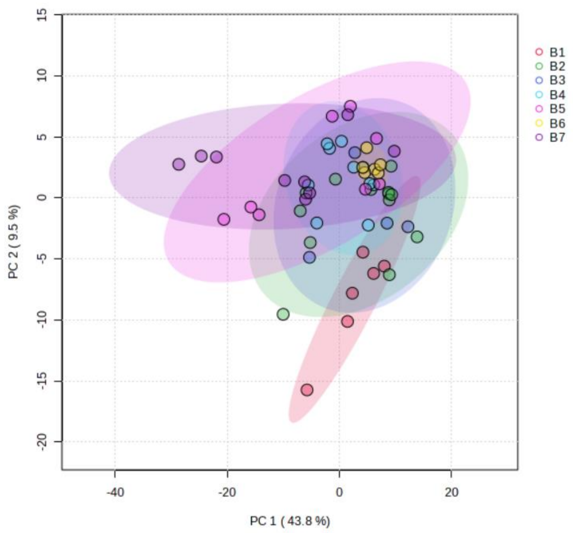

Fig S1 Principal component analysis scores plot indicating absence of batch effects. *This plot visually confirms that the QC samples do not exhibit any significant between-batch or within-batch effects, demonstrating consistent instrumental response and stable column performance throughout the analysis*

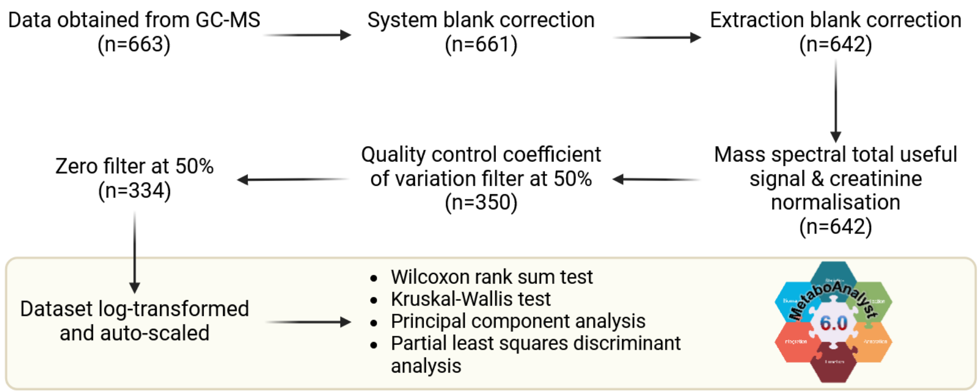

Fig S2 Data cleanup process. *This figure illustrates the cleanup process, including blank corrections, normalisation using mass spectral total useful signal and creatinine values, application of a 50% quality control coefficient of variation filter, and a 50% zero filter. The initial dataset of 663 compounds was refined to 334 compounds for further statistical analysis*

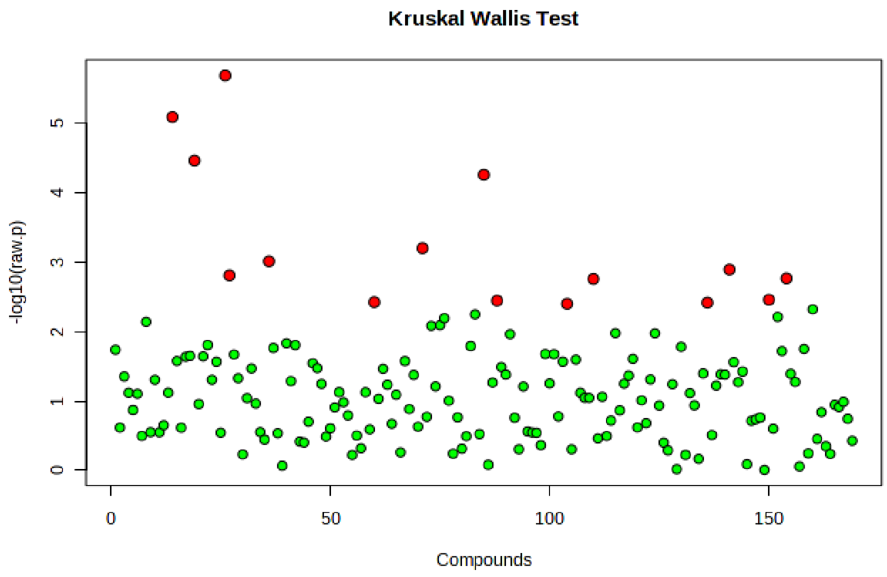

Fig S3 Kruskal-Wallis test visualisation. *This figure illustrates the outcomes of the Kruskal-Wallis test, where red dots represent statistically significant metabolites, and green dots denote metabolites not found to be significant*

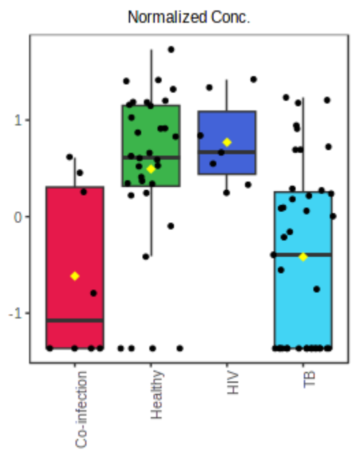

2,3-Butanediol

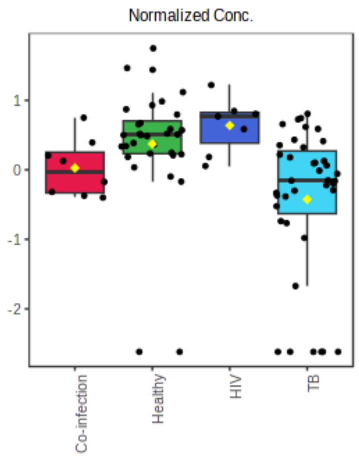

2,3-Dihydroxybutanoic acid

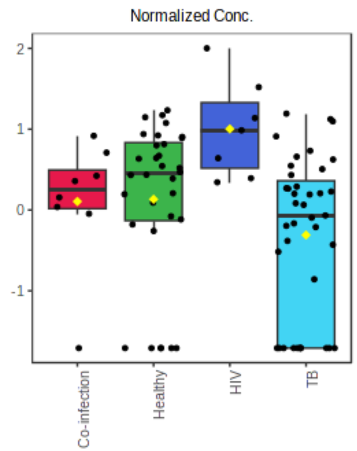

2-Deoxyribolactone

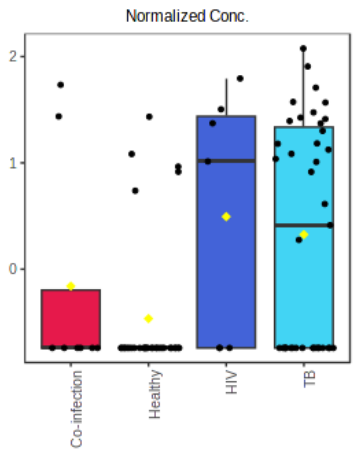

2-Methylene-butane-1,4-diol

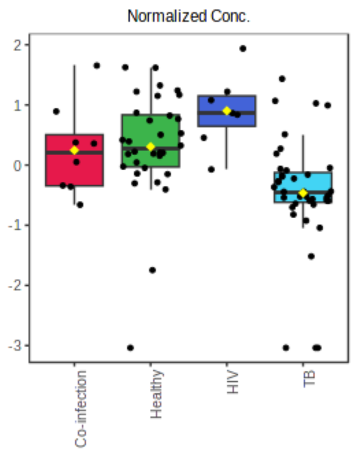

3-Hydroxy-2-methylpropanoic acid

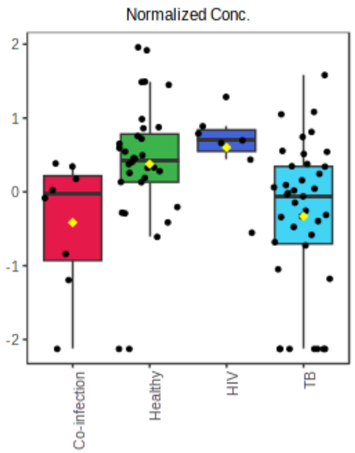

Citric acid

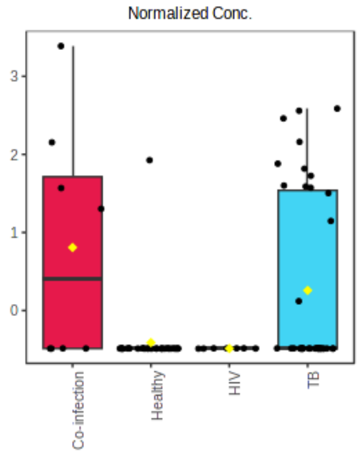

Diaveridine

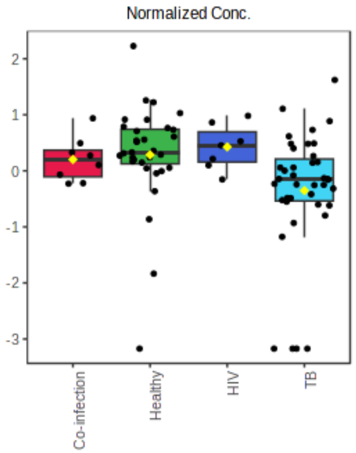

Erythritol

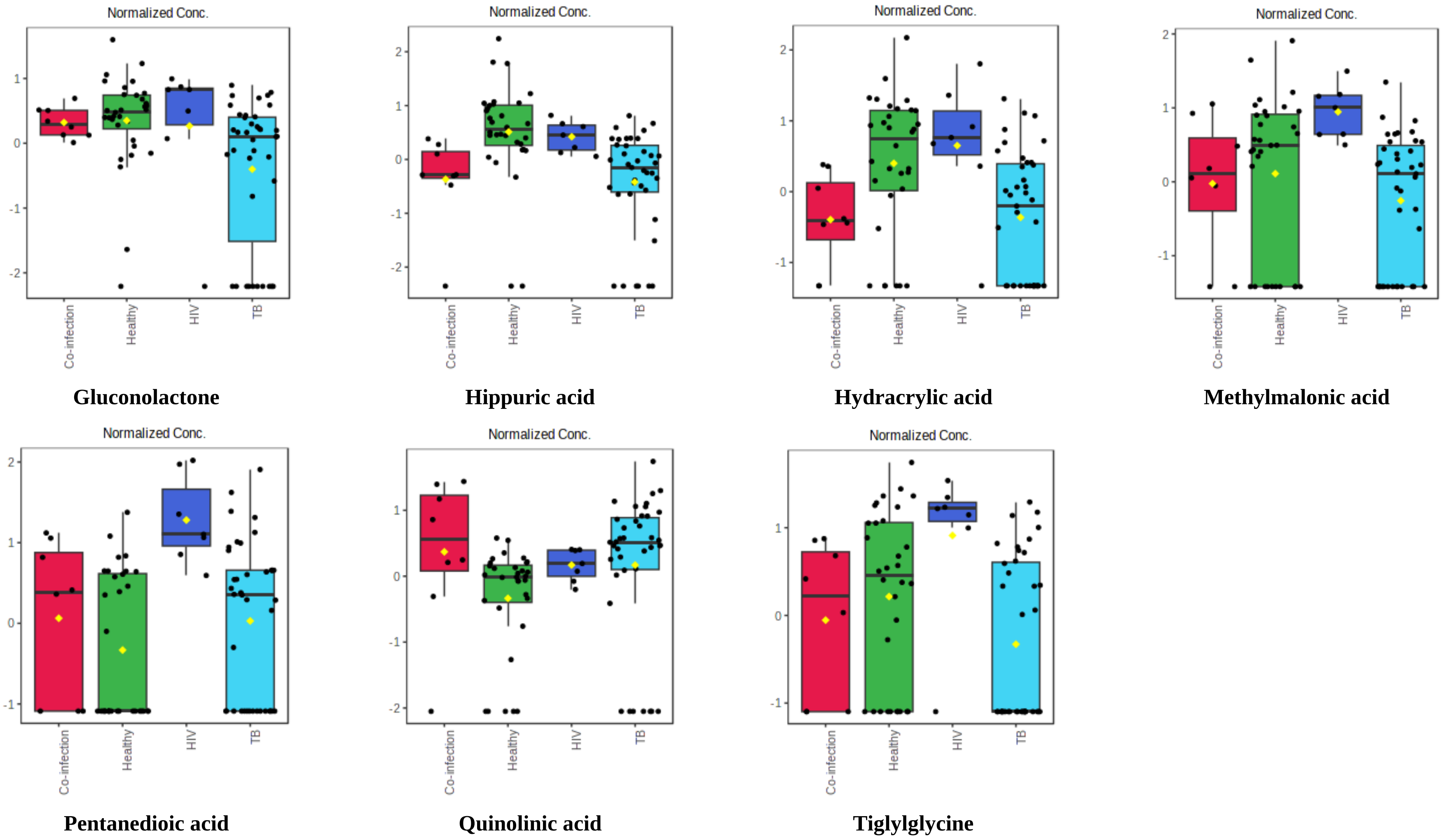

Fig S4 Boxplots of significant metabolites across different groups. *This image depicts individual samples (black dots), group averages (yellow dots), and group mean concentrations (horizontal lines within boxes)*

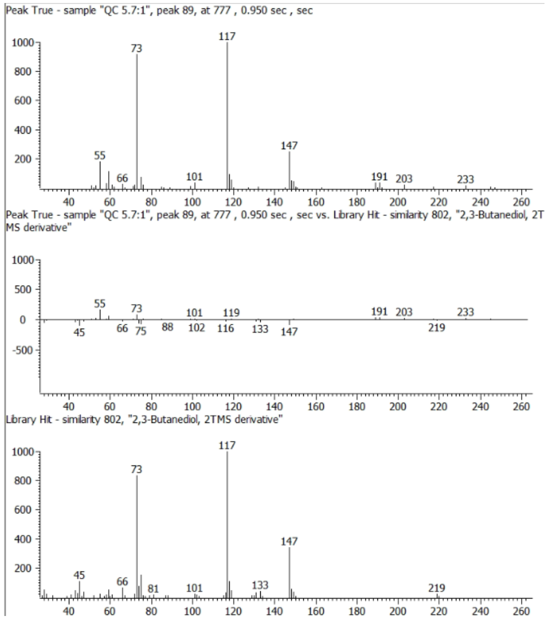

2,3-Butanediol

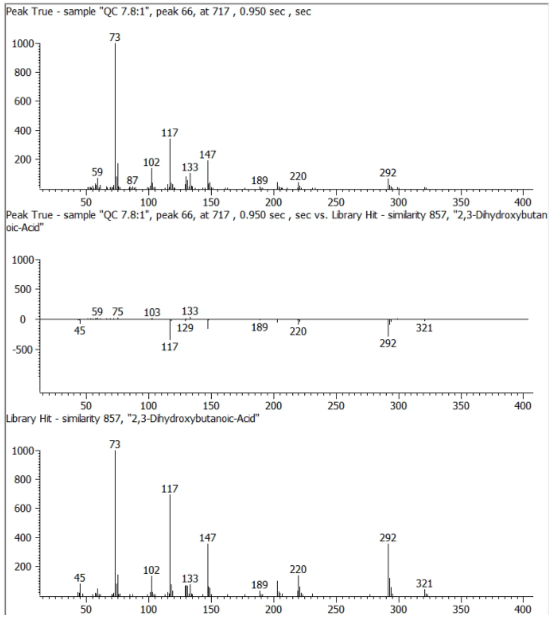

2,3-Dihydroxybutanoic acid

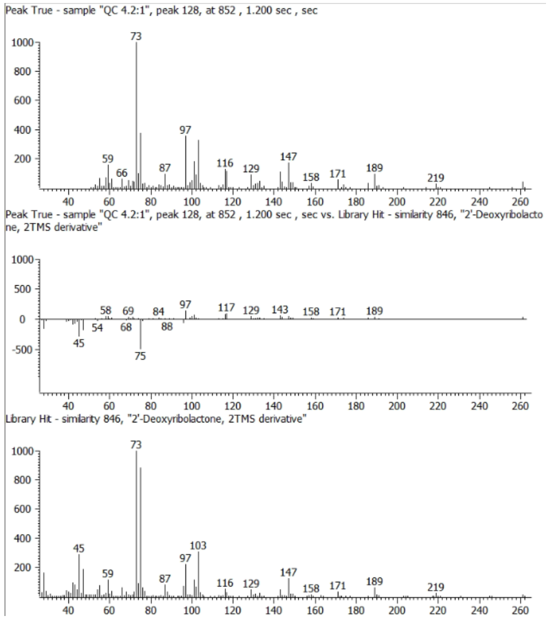

2-Deoxyribolactone

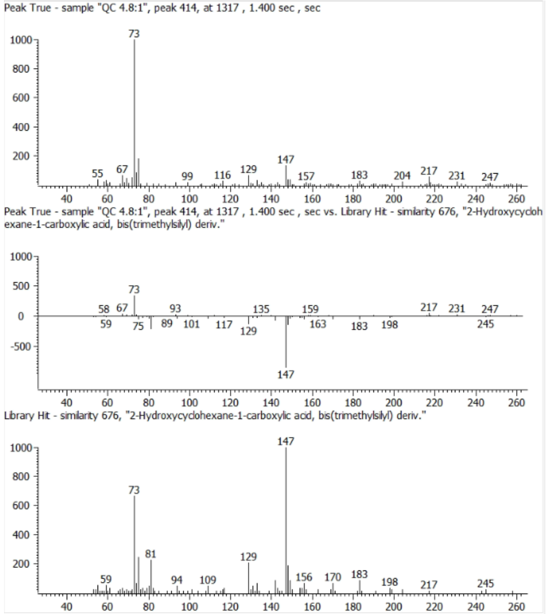

2-Hydroxycyclohexane-1-carboxylic acid

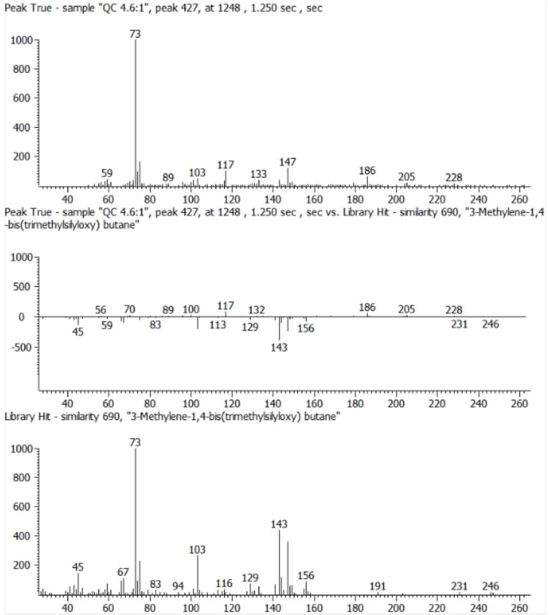

2-Methylene-butane-1,4-diol

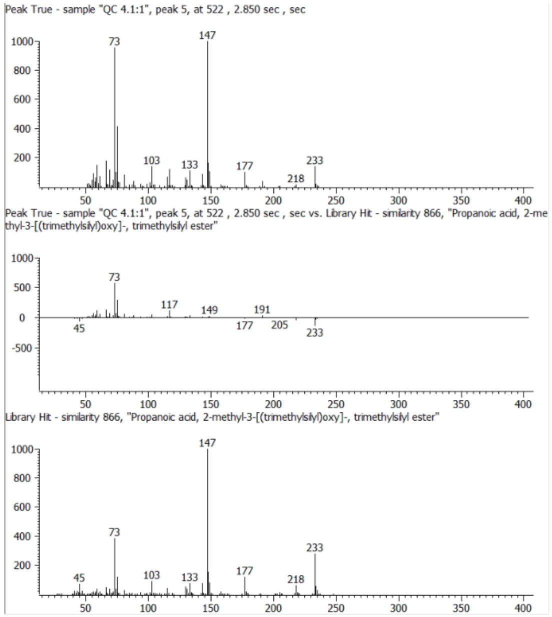

3-Hydroxy-2-methylpropanoic acid

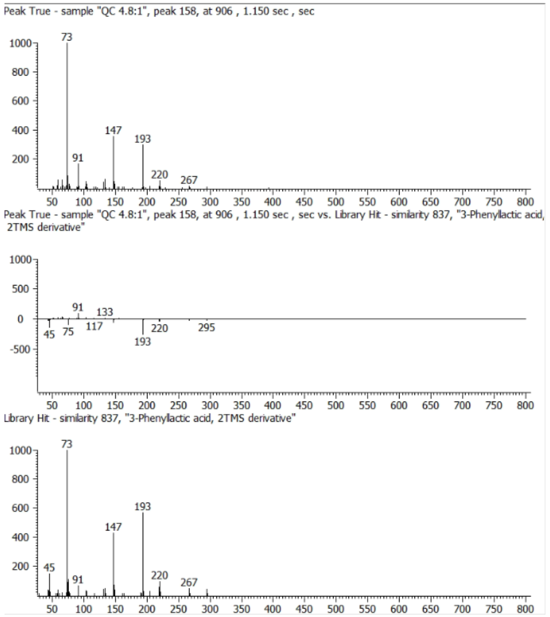

3-Phenyllactic acid

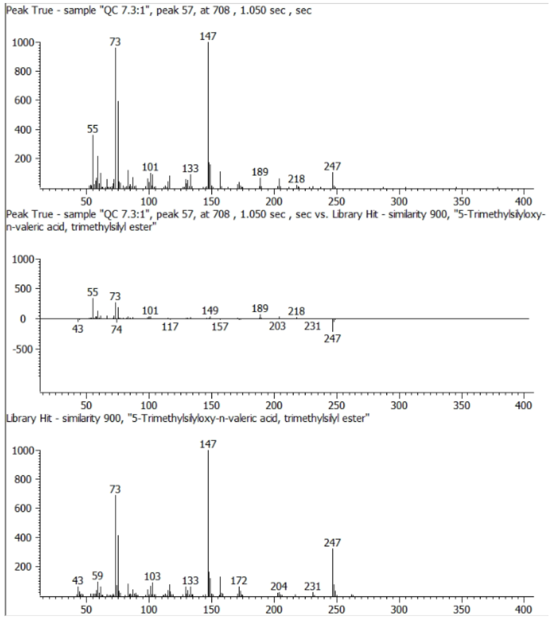

5-Hydroxyvaleric acid

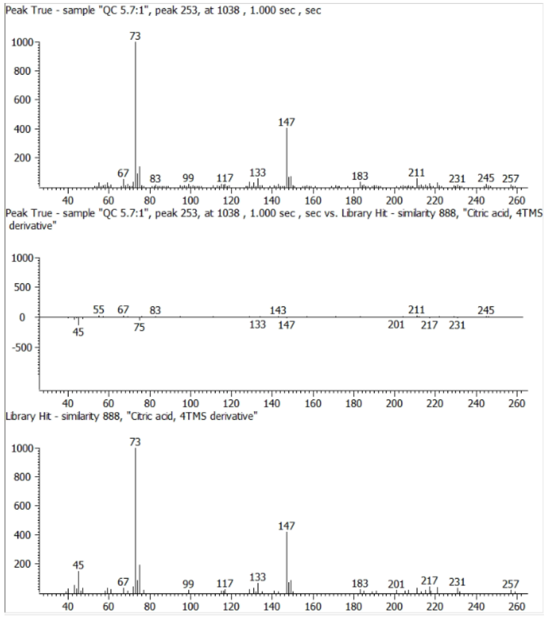

Citric acid

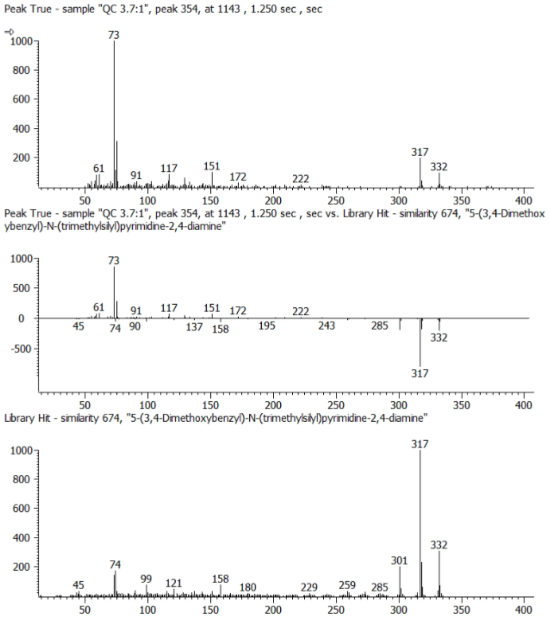

Diaveridine

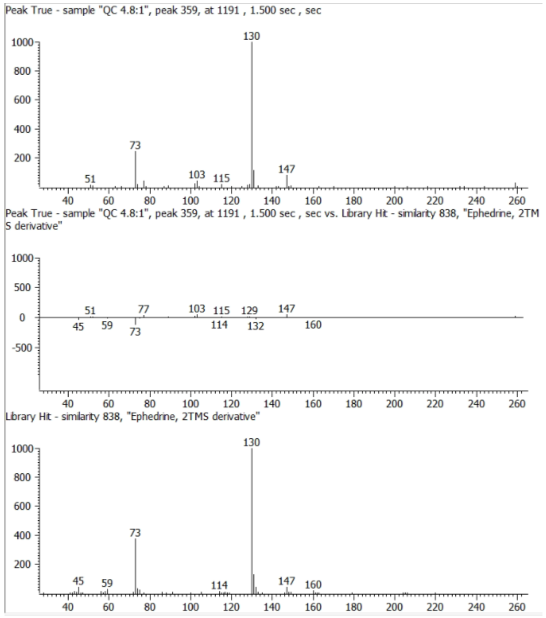

Ephedrine

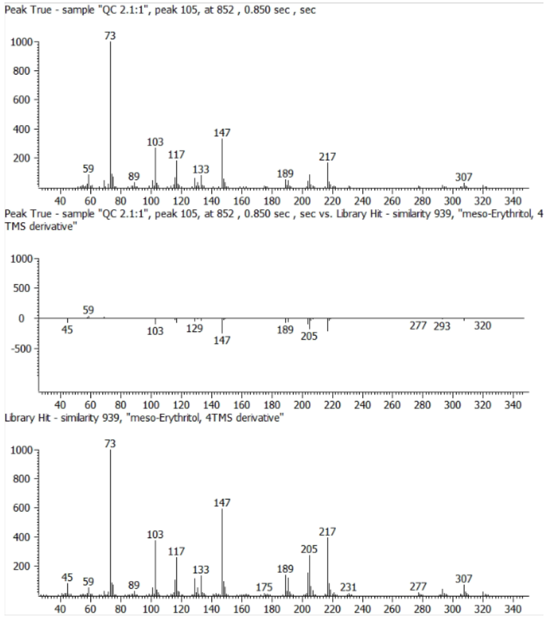

Erythritol

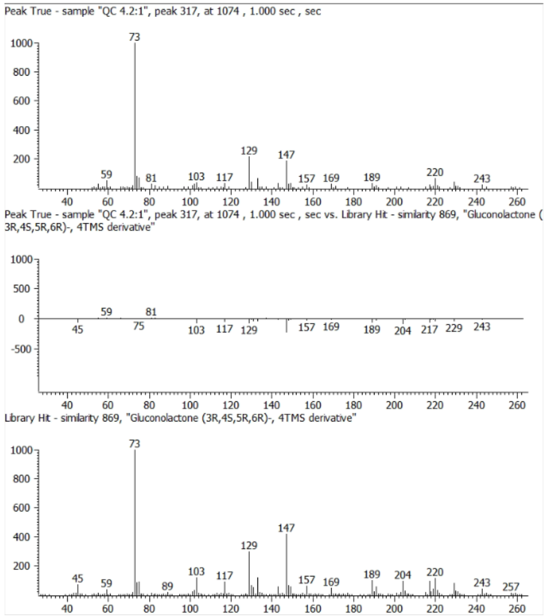

Gluconolactone

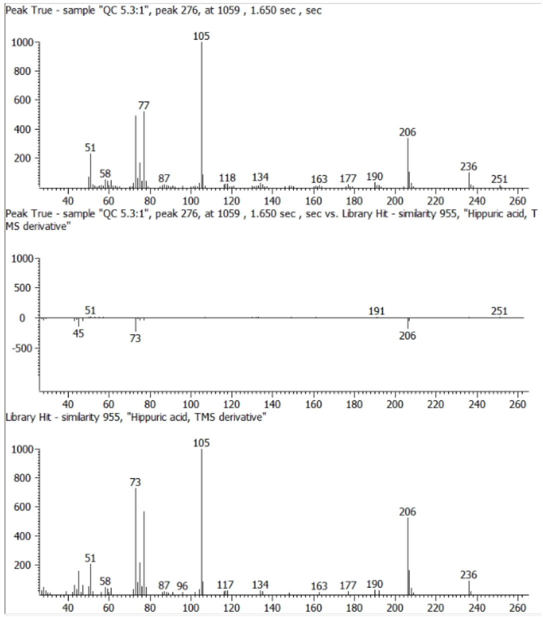

Hippuric acid

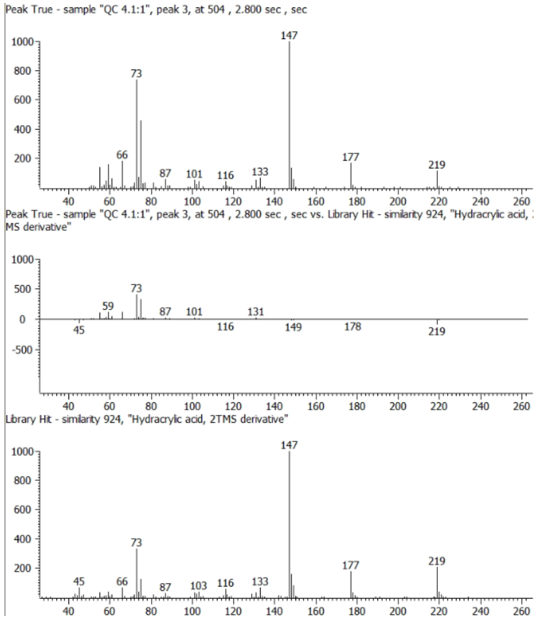

Hydracrylic acid

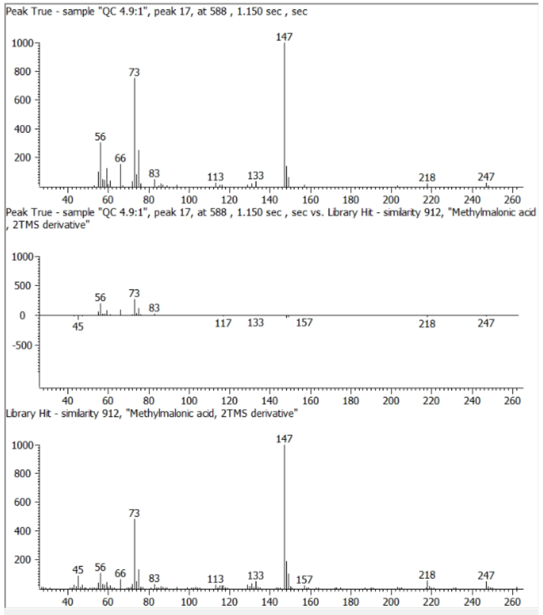

Methylmalonic acid

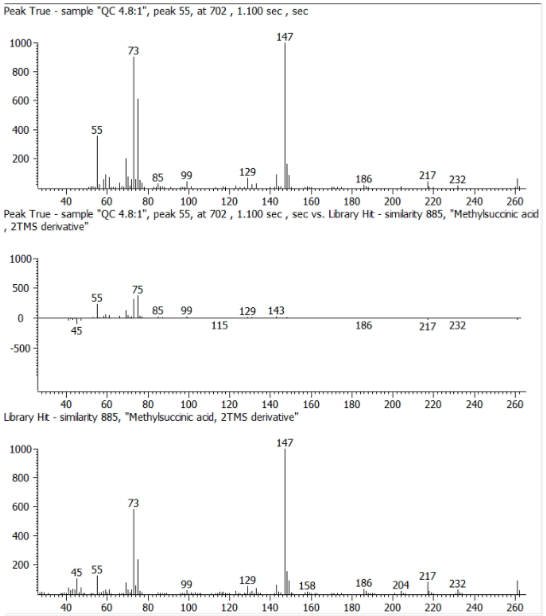

Methylsuccinic acid

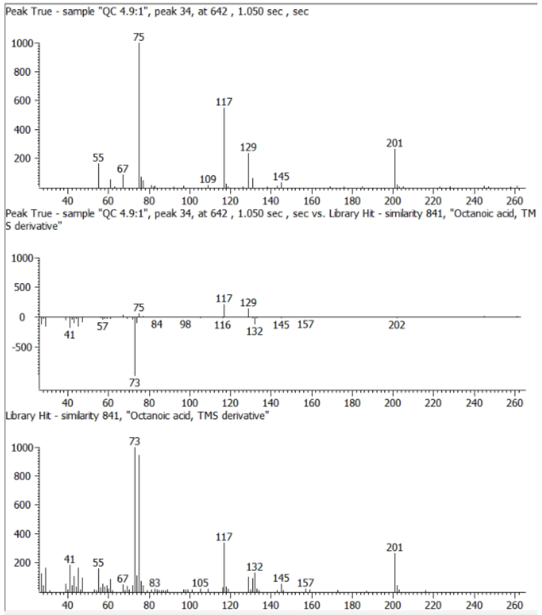

Octanoic acid

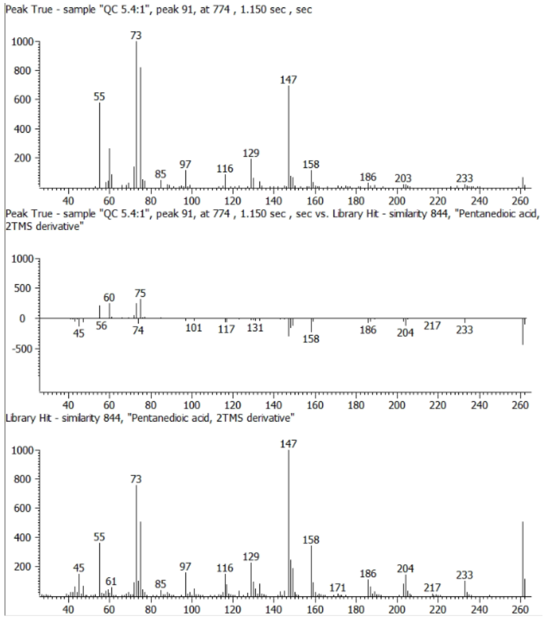

Pentanedioic acid

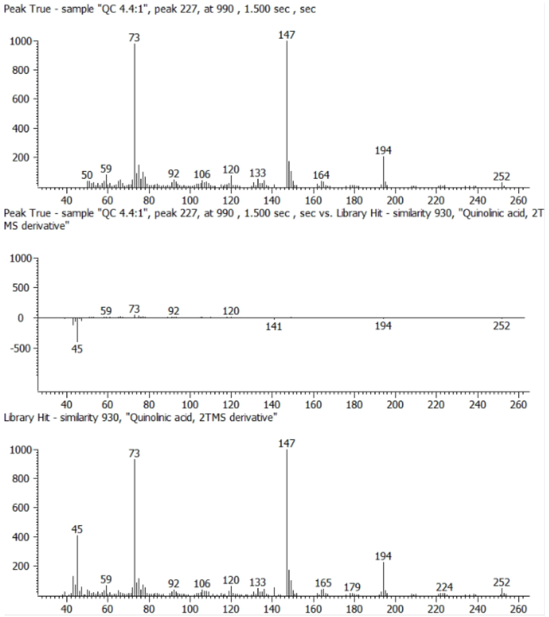

Quinolinic acid

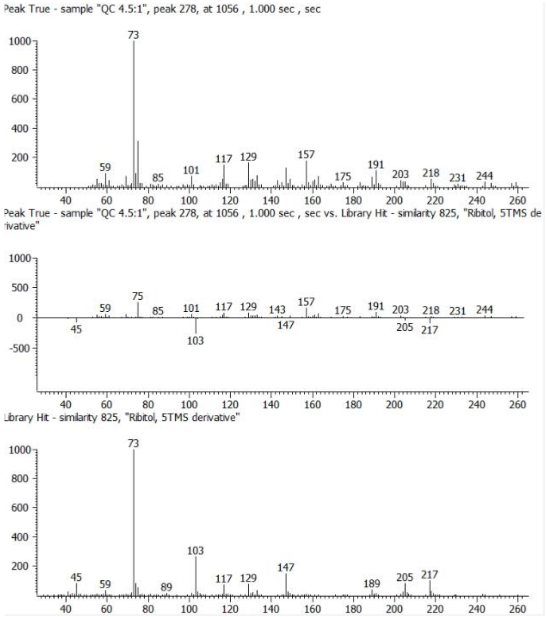

Ribitol

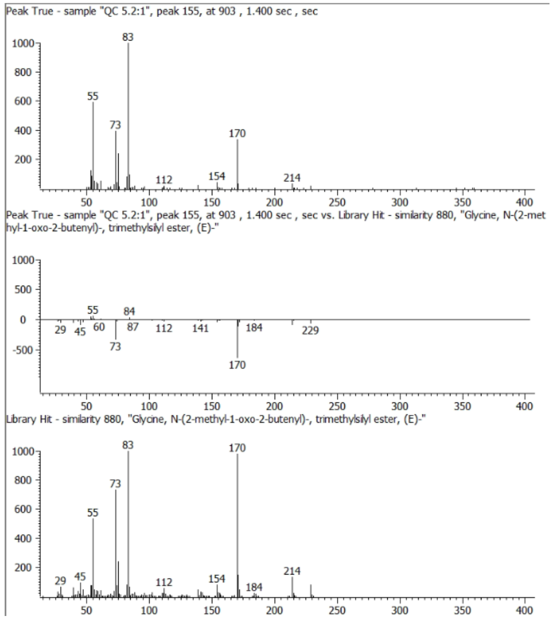

Tiglylglycine

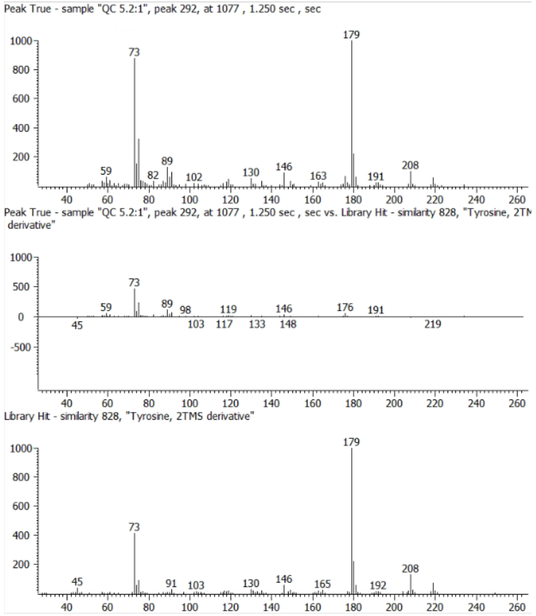

Tyrosine

Fig S5 Library spectral matching of all significant differential metabolites. *For each indicated compound, the top image depicts the spectra of the metabolite as detected in a sample, the middle image illustrates differences between the sample and the library, while the bottom image shows the library match*

PCA results: **PLSDA results:**

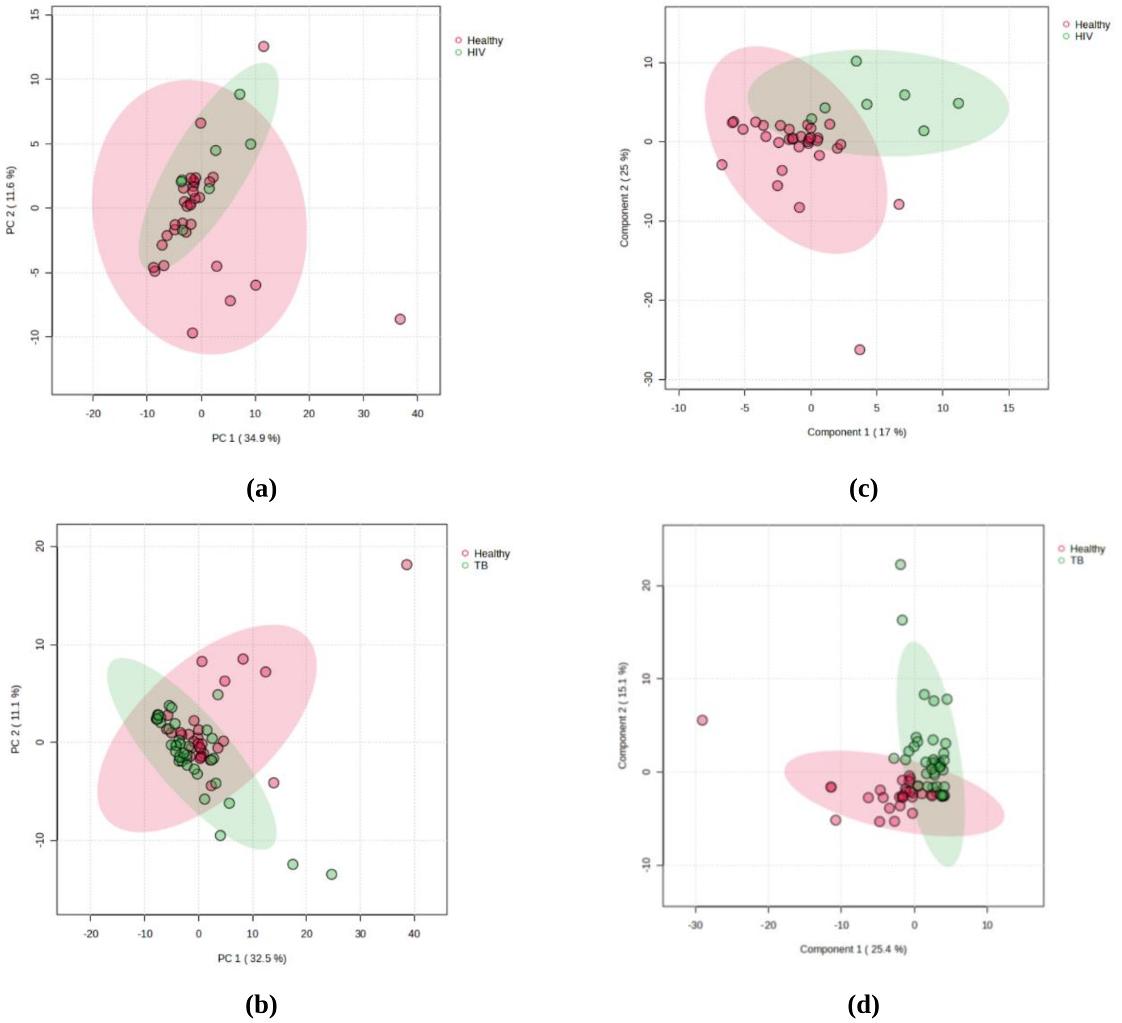

Fig S6 Comparative analysis using PCA and PLS-DA. *This figure illustrates the group comparisons using PCA for inherent data structure visualisation, and PLS-DA for group classification to emphasise the discriminative features between the groups. Specifically, panel (a) shows the PCA of HIV-positive only individuals compared to healthy controls, highlighting differences in their metabolic profiles. Panel (b) presents the PCA for TB-positive only individuals versus healthy controls. Panel (c) illustrates the PLS-DA for HIV-positive only individuals compared to healthy controls, emphasising discriminative features between these groups. Finally, panel (d) shows the PLS-DA for TB-positive only individuals versus healthy controls*

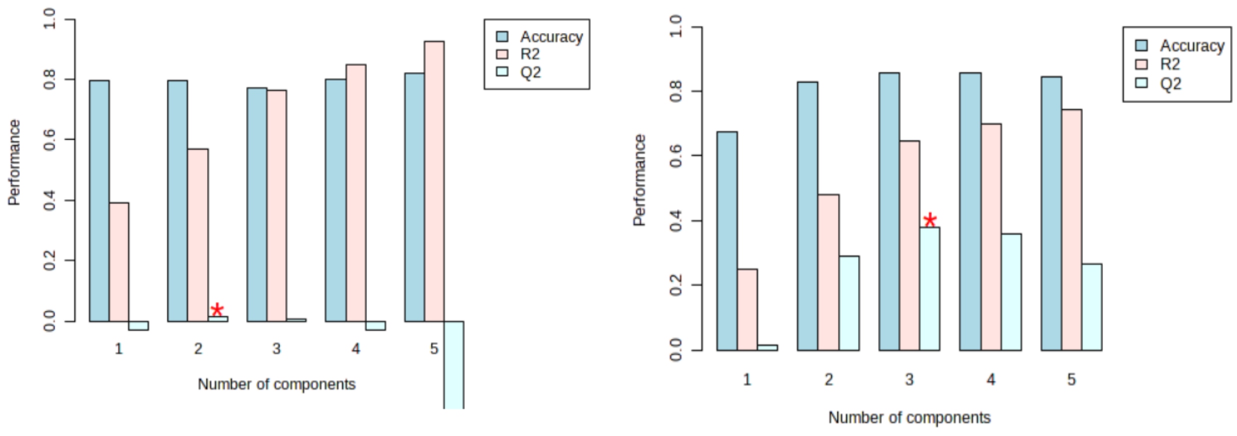

(a) **(b)**

Fig S7 Cross-validation test results. *Here, (a) displays the cross-validation tests for the HIV-positive only group versus healthy controls, and (b) shows the TB-positive only group versus healthy controls*

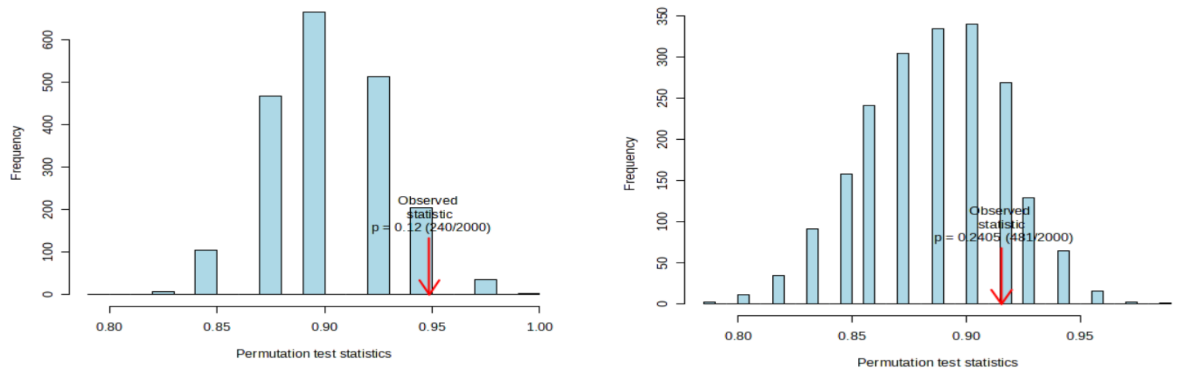

(a) **(b)**

Fig S8 Permutation test results. *Permutation tests results for the (a) HIV-positive only group versus healthy controls and the (b) TB-positive group only versus healthy controls*

Table S1: Significant metabolites identified by the Kruskal-Wallis test.

	**p-value**	**FDR (0.05)**	**Group with highest average concentration**
Hippuric acid	<0.001	<0.001	HIV-/TB-
3-Hydroxy-2-methylpropanoic acid	<0.001	0.001	HIV+/TB-
2,3-Butanediol	<0.001	0.002	HIV+/TB-
2,3-Dihydroxybutanoic acid	<0.001	0.002	HIV+/TB-
Quinolinic acid	0.001	0.022	HIV+/TB+
Citric acid	0.001	0.028	HIV+/TB-
Diaveridine	0.002	0.030	HIV+/TB+
Hydracrylic acid	0.001	0.030	HIV+/TB-
Erythritol	0.002	0.030	HIV+/TB-
Pentanedioic acid	0.002	0.030	HIV+/TB-
2-Deoxyribolactone	0.004	0.045	HIV+/TB-
2-Methylene-butane-1,4-diol	0.004	0.045	HIV+/TB-
Gluconolactone	0.004	0.045	HIV-/TB-
Tiglylglycine	0.004	0.045	HIV+/TB-
Methylmalonic acid	0.004	0.045	HIV+/TB-

Abbreviations: FDR: false discovery rate.

Table S2: Pairwise Comparison of Metabolite Levels Among Different Cohort Groups.

Note: Five pairwise comparisons were conducted to determine specific differences between groups, with a p-value threshold set using a false discovery rate (FDR) of 0.05 for significance. Listed below are the metabolites with significant differences observed.

**Group Comparison**	**Metabolite**	**P-value**	**FDR**	**Trend**
HIV-positive vs. Healthy controls	Pentanedioic acid	0.0002	0.034	↑ in HIV+/TB-
TB-positive vs. Healthy controls	See detailed list below*	-	-	Various trends noted
Co-infection vs. Healthy controls	None detected	-	-	No significant metabolites
Co-infection vs. HIV-positive	None detected	-	-	No significant metabolites
Co-infection vs. TB-positive	None detected	-	-	No significant metabolites

**Detailed List of Significant Metabolites for TB-positive (HIV-/TB+) vs. Healthy Controls (HIV-/TB-):*

	**P-value**	**FDR**	**Trend in HIV-/TB+ group**
Hippuric acid	<0.001	<0.001	↓
3-Hydroxy-2-methylpropanoic acid	<0.001	0.001	↓
2,3-Dihydroxybutanoic acid [2]	<0.001	0.002	↓
2,3-Butanediol	<0.001	0.004	↓
Quinolinic acid	<0.001	0.004	↑
Erythritol	0.001	0.014	↓
2-Methylene-butane-1,4-diol	0.001	0.014	↑
Gluconolactone	0.001	0.022	↓
Hydracrylic acid	0.001	0.022	↓
2-Hydroxycyclohexane-1-carboxylic acid	0.001	0.023	↑
Citric acid	0.002	0.024	↓
Diaveridine	0.002	0.029	↑
Octanoic acid	0.003	0.031	↓
Ribitol	0.003	0.031	↓
Tyrosine	0.003	0.031	↑
5-Hydroxyvaleric acid	0.003	0.031	↓
3-Phenyllactic acid	0.006	0.047	↑
Biphenyl (non-derivatized; potential artefact)	0.006	0.047	↑
2,3-Dihydroxybutanoic acid [1]	0.006	0.047	↓
Methylsuccinic acid	0.005	0.047	↓
Ephedrine	0.006	0.048	↑

Abbreviations: FDR: false discovery rate.
